# A Review of the Conservation Status of Shorebirds in Mongolia

**DOI:** 10.3390/ani14121752

**Published:** 2024-06-10

**Authors:** Sundev Gombobaatar, Dorj Ususkhjargal, Reuven Yosef

**Affiliations:** 1 Biology Department, School of Arts and Sciences, National University of Mongolia and Mongolian Ornithological Society, Ikh Surguuliin Gudamj 1, Ulaanbaatar 210646A, Mongolia; mongoliabirds@gmail.com; 2 Eilat Campus, Ben Gurion University of the Negev, Eilat 8810201, Israel

**Keywords:** waders, shorebirds, Mongolia, review

## Abstract

**Simple Summary:**

We conducted the first extensive review of shorebird species in Mongolia, examining their ecological status, population trends, threats, and conservation efforts. Mongolia is home to 62 shorebird species, including 6 globally threatened ones like the Sociable Lapwing and Siberian Sandplover. The country plays a crucial role in the breeding and migration of these birds, especially those at risk. While northern regions boast higher species diversity, threats like habitat loss and pollution endanger many species. Despite these challenges, Mongolia has general conservation measures in place and participates in international conventions. However, specific conservation plans for shorebirds are lacking, highlighting the need for more targeted efforts to protect these vulnerable species.

**Abstract:**

We present the first comprehensive review of 62 migratory shorebird species in Mongolia, covering their ecological status, IUCN assessments at regional or national levels, population trends, threats, and conservation measures. Mongolia hosts a total of 62 shorebird species from twenty-two genera and seven families, with six species classified as globally threatened: the Critically Endangered Sociable Lapwing, the Endangered Siberian Sandplover, the Far Eastern Curlew, the Great Knot, and the Vulnerable Sharp-Tailed Sandpiper. Both national and global IUCN Red List assessments highlight Mongolia’s significance as a breeding and passage migrating site for globally threatened and Near-Threatened shorebirds. Species richness is higher in northern regions compared to the south, with the highest diversity found in areas with complex aquatic ecosystems. Global population trends indicate a decline in 61% of species, with 18% remaining stable, 16% of unknown status, and 5% increasing. At the national level, most species are stable (61%), 34% status is unknown, and 5% are decreasing. Anthropogenic-induced threats, including habitat loss and degradation, pollution, disturbance, and harvesting, pose significant risks to 69% of species, while natural disasters affect 11%. Additionally, 8% of species are impacted by accidental mortality and intrinsic factors, and 5% by changes in native species. Despite these threats, no specific conservation action plans exist for shorebirds in Mongolia. However, general conservation measures are in place, such as environmental and fauna protection laws, regulations on foreign trade in endangered species, and the establishment of protected areas under governmental resolutions. Mongolia also participates in international conventions like the Convention on Biological Diversity (CBD), Ramsar, and Migratory Species (CMS), and has developed national red lists, red books, and publications such as “A Summary Conservation Action Plan for Mongolian Birds”, “Important Bird Areas” to support conservation efforts.

## 1. Introduction

Shorebirds, also known as waders, are members of the order Charadriiformes, which exhibit considerable species diversity, second only to passerines in terms of families and species OK [1]. The collective term “shorebirds” originates from their primary habitats, where the majority of species spend at least a portion of their life cycle, primarily on open tidal flats and shorelines of various wetlands, encompassing inland marshlands, rivers, lakes, lagoons, and artificial wetlands [2].

Globally, the taxonomy and phylogeny of shorebirds have received considerable attention, resulting in the identification of 391 species belonging to 19 families within three suborders: Charadrii, Scolopaci, and Lari. The Charadrii suborder comprises several families, including Burhinidae (10 species), Pluvianellidae (1), Chionidae (2), Pluvianidae (1), Charadriidae (69), Recurvirostridae (10), Ibidorhynchidae (1), and Haematopodidae (12). The Scolopaci suborder includes families such as Rostratulidae (3), Jacanidae (8), Pedionomidae (1), Thinocoridae (4), and Scolopacidae (98). Additionally, families like Turnicidae (18), Dromadidae (1), Glareolidae (17), Laridae (103), Stercorariidae (7), and Alcidae (25) are also recognized [2,3].

For the most recent analyses of taxonomy and phylogeny, all species within the families of the Charadri and Scolopaci suborders, including 17 species of Glareolidae, are classified as shorebirds, excluding Laridae (https://birdnet.org/2024/01/08/2023-annual-report/ accessed on 11 December 2023). This classification scheme is adhered to in this review paper. Among the 391 identified species [2,3], 237 species belong to two different suborders, including Glareolidae.

The most recent comprehensive list of shorebirds in Mongolia was compiled in 2008 [4]. This study marks the first endeavor to update the list, assess the ecology and conservation status, analyze threats and population trends, and review conservation measures. Following the IOC Bird List compiled [3], we listed 62 species of shorebirds belonging to 22 genera and 7 families. All shorebird species in Mongolia are migratory, except for the Solitary Snipe (*Gallinago solitaria*).

Considering multiple references [5,6,7,8,9,10,11,12], alongside our own data on shorebirds, the status analysis revealed that among shorebirds in Mongolia, 28% (24 species) are classified as Breeding Visitors, 56% (48 species) as Passage Migrants, 1% (1 species) as Partial Migrants, and 15% (13 species) as Vagrants. Of the 62 assessed species [10], 68% (42 species) are categorized as regionally or nationally Least Concern, 6% (4 species) as Data-Deficient, and 24% (15 species) as Not Applicable. Mongolia serves as a stronghold for globally threatened shorebirds, including the Critically Endangered Sociable Lapwing (*Vanellus gregarius*), Endangered Siberian Sandplover (*Charadrius/Anarhynchus mongolus*), Far Eastern Curlew (*Numenius madagascariensis*), Great Knot (*Calidris tenuirostris*), and Vulnerable Sharp-Tailed Sandpiper (*Calidris acuminata*). Additionally, the Asian Dowitcher (*Limnodromus semipalmatus*) is assessed as globally Near-Threatened and regionally or nationally Vulnerable.

This paper utilizes baseline data on species richness, population trends, and threats from Gombobaatar et al. [10], which is the most recent and reliable source for Mongolia shorebirds at present. Species richness of shorebirds in Mongolia is higher in the north compared to the south. The majority of shorebird population trends in Mongolia are stable at the national level, while a high proportion exhibit decreasing trends globally [10]. Human-induced factors such as habitat loss and degradation, pollution, disturbance, and harvesting pose significant threats to 69% of shorebird species, followed by natural disasters (11%), accidental mortality and intrinsic factors (8% each), and changes in native species (5%).

Wetlands in river valleys and lakes vary in vegetation composition based on geographical and topological characteristics, water body size, and shoreline vegetation. Dominant plant species in most wetlands include *Potamogeton* spp., *Sagittaria* spp., *Phragmites* spp., *Zannichellia* spp., *Najas* spp., *Batrachium* spp., and *Hippuris* spp. Gravel and sandy shores, bogs, and marshes with tall sedges and reeds at lakes and rivers play a crucial role for various shorebird species. Numerous migratory species utilize these habitats for resting, refueling, and passage, including plovers (Charadriidae), godwits (*Limosa* spp.), curlews (*Numenius* spp.), sandpipers (Scolopacidae), and stints (*Calidris* spp.). In the Gobi Desert, oases serve as critical resting and refueling sites for migrants like Black-Winged Stilt (*Himantopus himantopus*), Northern Lapwing (*Vanellus vanellus*), Pacific Golden Plover (*Pluvialis fulva*), and others, after their long journey from breeding to wintering grounds. However, a few species, such as Wood Sandpiper (*Tringa glareola*) and Pintailed Snipe (*Gallinago stenura*), prefer forested or slightly closed habitats during the breeding season, while Greater Sand (*Charadrius/Anarhynchus leschenaultii*) and Oriental Plovers (*Charadrius veredus*) favor dry open desert steppe for nesting [12].

There is a notable absence of legislation or legal frameworks specifically dedicated to the conservation of shorebirds in Mongolia, as evidenced by the information available at https://legalinfo.mn/mn (accessed on 20 April 2024). However, we provide an overview of actions and initiatives aimed at shorebird protection within the framework of national legislation, international conventions, establishment of protected areas, and other pertinent sources, such as the *Regional Red List for Mongolian Birds* [10], *Mongolian Red Book* [13,14], and *Important Bird Areas* [15].

Our overarching objective was to compile the most recent species list to assess the current status of shorebirds’ ecology and conservation, including threats and population trends. This information aims to facilitate the prioritization of conservation actions for the future. Additionally, our efforts seek to enhance global awareness and understanding of shorebirds while identifying research gaps to foster international cooperation in their conservation efforts.

## 2. Methods and Materials

### 2.1. Study Areas

Our comprehensive review of the ecological status and conservation efforts, distribution, population trends, and threats encompasses all regions of Mongolia, spanning an expansive area of 1.5 million km^2^. The capital city, Ulaanbaatar (47°55′7.67″ N, 106°55′3.33″ E), is centrally located. Mongolia, a landlocked East Asian country situated between Russia and China, boasts a total land area of 1,564,116 km^2^, ranking as the 18th largest and most sparsely populated nation globally [12].

Characterized by four distinct seasons, Mongolia experiences significant temperature fluctuations and low precipitation. Summers tend to be hot, while winters are extremely cold, with January averages plummeting to as low as −30 °C. The annual mean temperature ranges between −8 °C and 6 °C, with annual mean precipitation varying from 50 mm in the Gobi Desert to 400 mm in northern mountain ranges (http://www.tsag-agaar.gov.mn; accessed on 20 April 2024).

Approximately 30% of Mongolia’s population resides in rural areas, where traditional nomadic and semi-nomadic lifestyles prevail, with the majority owning the nation’s 67,068,490 head of livestock (https://www.nso.mn accessed on 20 April 2024). The country boasts diverse vegetation across various natural zones and ecosystems, namely high mountains, mountain taiga forest, forest steppe, steppe, desert steppe, and Gobi Desert [16,17]. However, Chimed-Ochir et al. [18] classified six distinct ecosystems in Mongolia, namely high mountains, forest, steppe, desert, aquatic, and patch.

**Figure 1:**
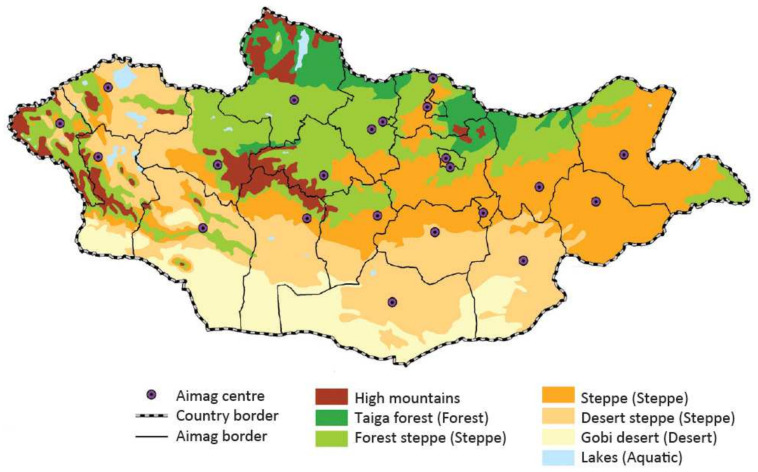
The geophysical ecosystems of Mongolia.

Inland wetlands, encompassing rivers, freshwater and saline lakes, springs, creeks, ponds, marshes, lake shores, lagoons, river deltas, Gobi Desert oases, and artificial ponds, are distributed across three major river basins: the Central Asian Internal Drainage Basin, the Arctic Ocean, and the Pacific Ocean [19] (Figure 1).

These wetlands serve essential functions in the breeding, foraging, resting, roosting, and refueling activities of both breeding and migratory shorebirds.

### 2.2. Data Collection, Analysis of Species Composition, Ecology, and Conservation Status

The latest update on the shorebirds of Mongolia, conducted by Gombobaatar [12], adhered to the taxonomy and classification system outlined by the IOC, as detailed in the work by Gill et al. [3]. Primary sources of data regarding the status of shorebirds within Mongolia included studies by Fomin and Bold [7], Dawaa et al. [8], Gombobaatar et al. [10], and Gombobaatar [12]. Notably, most breeding species exhibit dual statuses due to migrating individuals traversing breeding and stopover sites in Mongolia during spring or southward and autumn or northward migrations.

The status of the ecology of each species is delineated as follows:

- Breeding Visitor (BV): Species that breed in Mongolia but migrate, typically to more southern latitudes;
- Passage Migrant (PsM): Species that breed at higher latitudes and pass through Mongolia during migration;
- Partial Migrant (PrM): Species that breed in Mongolia and winter in small numbers.

The concept of “partial migration” is prevalent, characterized by within-population variation in migratory tendency, wherein only a fraction of the population migrates [20].

Additionally, species categorized as Vagrant (VA) have been recorded fewer than ten times in Mongolia. Non-Breeding Summer Visitor (NB) species, lacking sufficient data and information on their occurrence in Mongolia during the summer, were excluded from our analysis.

A rating score ranging from 1 to 10 (VA1-10) was utilized based on documented records for each species. Abundance Rating was categorized as follows:

- Scores of 1 and 2: Rare to Scarce;
- Scores of 3 and 4: Fairly Common to Common;
- Scores of 5 and 6: Very Common to Abundant.

This rating system provides a general indication of the abundance of each species in the country, with ratings such as 1/2, 2/3, 3/4, 4/5, and 5/6 signifying an abundance bordering two scores [12].

Furthermore, the IUCN global population assessment for each species was referenced from the “2004 IUCN Red List of Threatened Species” [21]. National assessments of Mongolian birds were initially published in the *Regional Red List for Mongolian Birds* [10], utilizing the “IUCN Red List Categories and Criteria: Version 3.1” [22], and the “Guidelines for Application of IUCN Red List Criteria at Regional Levels: Version 3.0” [21]. Global assessments of each species were cited from Birdlife International [23] and IUCN [24] (Appendix A).

### 2.3. Data Collection, Analysis of Distribution, and Species Richness

**Figure 2:**
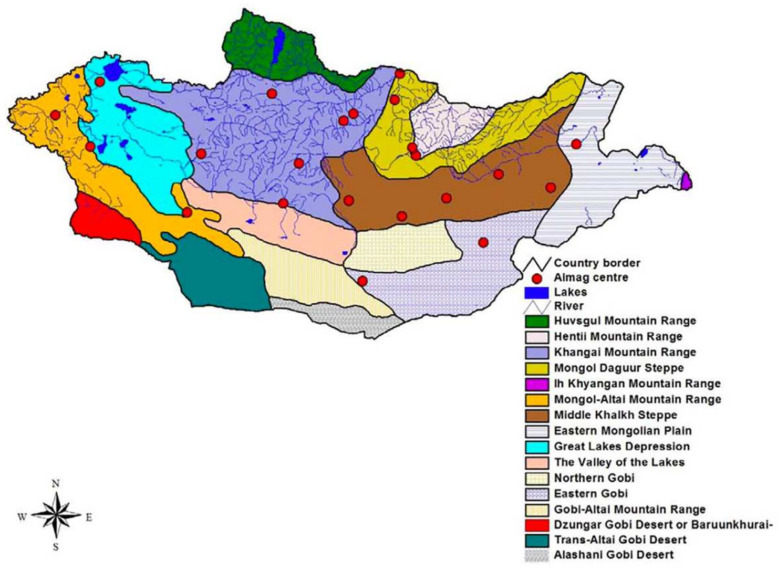
The geobotanical subdivisions of Mongolia.

Fomin and Bold [7] elucidated the distribution of birds in Mongolia utilizing botanical–geographical subdivisions guided by the principles outlined by Yunatov [16], Grubov [25,26,27], Ulziikhutag [28], and [17] (Figure 2).

Fomin and Bold [7] utilized subdivision for a broad delineation of breeding and non-breeding areas of birds in Mongolia. Following their approach and incorporating descriptions provided by Fomin and Bold [7], along with the general distribution map by Dawaa et al. [8], we outlined a preliminary distribution polygon for each species, represented in monochromatic colors on maps. Subsequently, unsuitable breeding and non-breeding habitats were excluded from the original distribution polygons using MaxEnt programs, which estimate probability distributions by calculating the potential distribution probability of each species [29].

The demarcation of habitats was informed by MaxEnt (Maximum Entropy Modeling 3.3.0) analyses and .shp files or subsets detailing various habitats, natural zones, and features such as bodies of water ranging from small creeks to large rivers and lakes, as well as alpine and subalpine meadows; high mountains with varying altitudes; low hills; and specific plant communities including Caragana, reeds, and coniferous and deciduous trees. These .shp files for GIS maps (version 10.8) were initially developed by several organizations, including WWF, the Mongolian Ornithological Society, the GIS Laboratory of the National University of Mongolia, and the Russian and Mongolian Academy of Sciences [17].

Utilizing these .shp files and MaxEnt analyses, we were able to delineate general and potential habitats for each shorebird species across the entire border of Mongolia. We incorporated coordinates for every species’ distribution record onto the distribution map using small dots and polygons. Due to the inherent uncertainty of breeding records and the availability of limited information, a rigorous separation of breeding habitats proved challenging for the illustration of the maps [10].

Upon drawing the distribution maps for each species, we overlaid all species’ distribution maps using ArcMap software (10.8.2) at the laboratory of the National University of Mongolia to estimate species richness while integrating various parameters such as protected areas [19] and botanical–geographical subdivisions [16,25,26,27,28].

### 2.4. Data Collection and Analysis of Population Trends

During the regional red list workshop on birds held in Ulaanbaatar, Mongolia, in 2011, experts assessed the population trends of 476 bird species in Mongolia, comparing them to the population data from 2000. This evaluation followed the approach and principles outlined by Birdlife International [10,30]. Baseline data on population trends for each shorebird species were obtained from various sources [5,6,7,8,9,10,11,12,31,32], and these data were extensively discussed among national and international experts during the workshop. Specifically focusing on shorebirds, we analyzed the population trends based on the data derived from this assessment and juxtaposed them with the global population trend, as reported by BirdLife International [23] and IUCN [24].

### 2.5. Data Collection and Analysis of Threats

Threat analysis plays a pivotal role in formulating effective conservation action plans for shorebirds in Mongolia in the near future. To achieve this, we employed the threat categories and matrix provided by BirdLife International [23] and IUCN [24]. During the 2011 workshop, national and international experts thoroughly examined the threats and potential threats facing all shorebird species in Mongolia. Baseline data on these threats were initially compiled from various sources [5,6,7,8,9,10,11,12,31,32] and referenced from the National Biodiversity Database, which contributed to the creation of the IUCN offline database [10].

We categorized threats into primary, secondary, and tertiary ranks, considering specific criteria and assigning scores accordingly. These criteria included the extent and severity of threats to the population in Mongolia, the ecological status of the species (e.g., Breeding Visitor, Passage Migrant, and Vagrant), conservation status based on the IUCN Red List categories at national and international levels, and population trends.

The primary rank of threats was assigned a score of “1”, and the scoring system was deliberated and finalized by national and international teams during the “Mongolian Bird Red List” workshop in 2011 [10]. Subsequently, with some modifications and additions to the data gathered in 2011, a new data table was created for this threat analysis (Appendix B).

In assessing the threat scores, we compared each threat identified and categorized by BirdLife International [23] against the ecology, conservation status, and population trends of each shorebird species (Appendix B). To determine any significant differences between threat categories and the rank of threats (primary, secondary, and tertiary), we conducted statistical analyses using SPSS software v269 and Open Source Statistics. Specifically, we employed ANOVA analysis, *t*-test (t), and the Kolmogorov–Smirnov test of normality (D). These analytical tools provided valuable insights into the relationships between threat categories and their respective ranks, facilitating a comprehensive understanding of the threats facing shorebird species in Mongolia.

### 2.6. Data Collection and Analysis of Conservation Actions

For generations, Mongolians have upheld traditions of protecting shorebirds and other wildlife, deeply rooted in their Buddhist faith. Legislative measures and governmental resolutions have been established to regulate biodiversity conservation and promote sustainable use, encompassing the protection of avian species. We meticulously referenced all pertinent laws and resolutions concerning migratory birds and other wildlife, drawing from the legislative framework of the Mongolian Government (https://legalinfo.mn; accessed on 10 April 2022).

In our review, we provide a concise overview of the current status of protected area networks in Mongolia, along with an analysis of the distribution range of each shorebird species within these protected areas. Additionally, we incorporate information from various international conventions, including the Convention on Biological Diversity, the Ramsar Convention, and the Convention on Migratory Species. Some data and insights regarding these conventions were derived from various sources [33].

Our study draws extensively from a diverse range of sources, including the database and published materials of the *Regional Red List for Mongolian Birds* [10]. We also reference the *Mongolian Red Book* [16,17] and the *Important Bird Areas* [18] to enrich our understanding and analysis of shorebird conservation in Mongolia.

## 3. Results

### 3.1. Species Composition, Ecology, and Conservation Status

**Figure 3:**
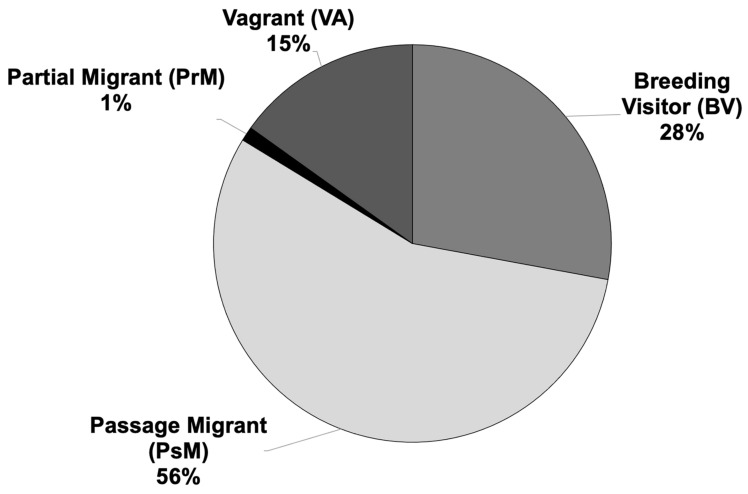
The ecological status of shorebirds in Mongolia.

In accordance with the IOC Bird List [3], our assessment has identified a total of 62 species of shorebirds, representing 22 genera and 7 families (see Appendix A). Among these species, 24 (28%) are classified as Breeding Visitors (BVs), 48 (56%) as Passage Migrants, 1 (1%) as Partial Migrant, and 13 (15%) as Vagrants (Figure 3). Given the migratory behavior inherent in all shorebird species in Mongolia, statistical analysis revealed no significant difference between each status (D = 0.219, *p* > 0.5). However, it is noteworthy that we documented only one species, the Solitary Snipe, which overwinters in small numbers in unfrozen open water areas of large rivers such as Khovd and Bukhmurun in the Central Asian Internal Drainage Basin in western Mongolia.

It is worth noting that due to some species exhibiting two different statuses, the total number of calculated species has increased from 62 to 86 (Figure 3, Appendix A).

Among the 62 assessed shorebird species in Mongolia, 1 species (2%) is classified as Vulnerable, 42 species (68%) as Least Concern, 4 species (6%) as Data-Deficient, and 15 species (24%) as Not Applicable. None were assessed as Critically Endangered, Endangered, and Near-Threatened categories. However, the majority of the species at the national level were of Least Concern (t = 1.503, *p* = 0.091). Notably, the Asian Dowitcher was assessed as nationally Vulnerable (VU) (refer to Figure 2).

**Figure 4:**
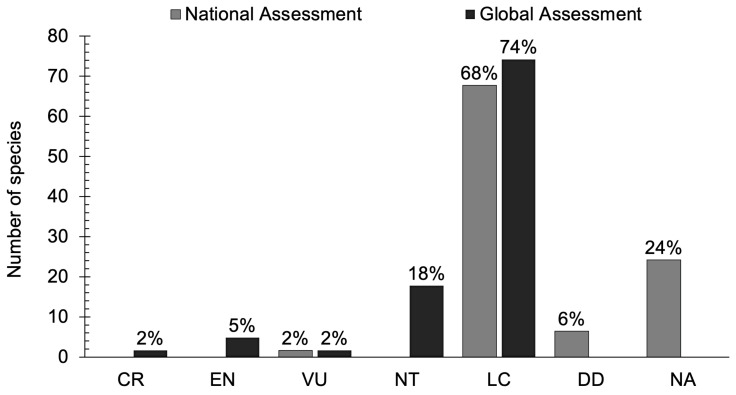
The national and global assessments of the IUCN Red List.

Additionally, Mongolia is home to six globally threatened bird species, namely the Critically Endangered Sociable Lapwing (2% of the global population), as well as the Endangered Mongolian/Siberian Sandplover, Far Eastern Curlew, Great Knot (5%), and Vulnerable Sharp-Tailed Sandpiper (2%). Furthermore, the Eurasian Oystercatcher (*Haematopus longirostris*), Northern Lapwing, Eurasian Curlew (*Numenius arquata*), Bar-Tailed Godwit (*Limosa lapponica*), Black-Tailed Godwit (*Limosa limosa*), Red Knot (*Calidris canutus*), Curlew Sandpiper (*Calidris ferruginea*), Red-Necked Stint (*Calidris ruficollis*), Buff-breasted Sandpiper (*Calidris subruficollis*), Asian Dowitcher, and Gray-Tailed Tattler (*Tringa brevipes*) are classified as Near-Threatened (NT) (18%) and Least Concern (LC) (74%) among the 62 assessed species [23]. There is no significant difference (t = 1.39, *p* = 0.106) between global IUCN categories among all assessed species occurring in Mongolia (Figure 4, Appendix A). The Asian Dowitcher is globally Near-Threatened and regionally or nationally threatened, making it a significant concern for Mongolia.

### 3.2. Distribution and Species Richness

The species richness of shorebirds in Mongolia exhibits a notable disparity between the north and south and the west and east regions, with greater diversity observed in the northern and eastern territories. This pattern is attributed to the higher habitat complexity present in the north and east, characterized by a diverse array of habitats, including various aquatic ecosystems.

Specifically, regions with high species richness include the geo-botanical divisions of the Mongol Daguur Steppe (mean 34.5 species, range 6–42), Eastern Mongolian Plain (especially around Buir Lake and the northeastern sector) (mean 37.8 species, 26–42), northern limit of Middle Khalkh Steppe (mean 28.4 species, min. 16, max. 42), and Great Lakes Depression (mean 23.7 species, 5–42). Some parts of the Ikh Khyangan Mountain Range, eastern Khangai Mountain Range, and western Khuvsgul Mountain Range (only Darkhad Depression) are secondary areas of high species richness.

**Figure 5:**
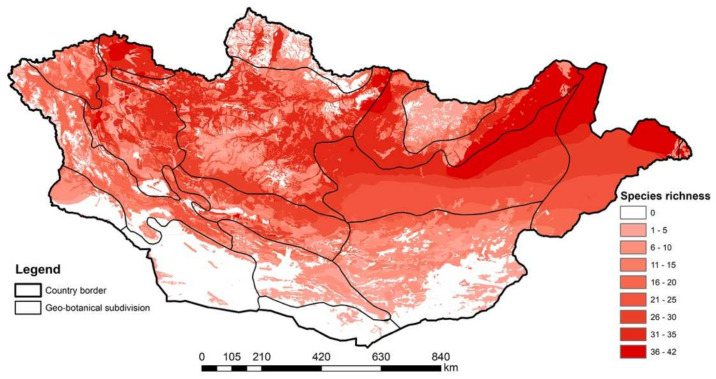
Avian species richness of Mongolia by geographic divisions.

Conversely, regions in the south, particularly the main high mountain ranges (Gobi-Altai, Mongol Altai, Khuvsgul, Khangai, and Khentii), desert steppe, and Gobi Desert areas, exhibit lower species richness. This disparity can be attributed to the limited availability of water resources in these arid landscapes (Figure 5).

### 3.3. Population Trends

**Figure 6:**
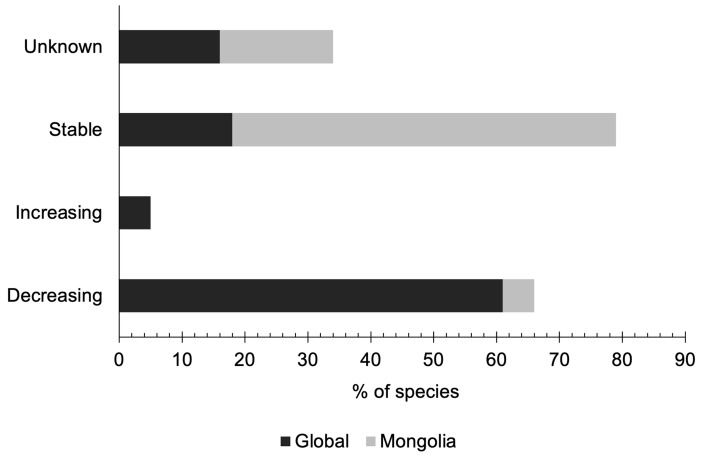
A comparison of the global and the Mongolian national assessment of population trends of shorebirds.

The global population trends of shorebirds in Mongolia indicate that the majority of species are experiencing a decline, with 38 species (61%) exhibiting a decreasing trend, followed by 11 species (18%) classified as stable, 10 species (16%) with unknown trends, and 3 species (5%) showing an increasing trend (refer to Figure 6).

At the national level, the population trend assessment reveals that the majority of shorebird species are classified as having a stable trend (38 species; 61%), followed by 21 species (34%) categorized as having an unknown trend, and only 3 species (5%) experiencing a decreasing trend (Figure 6).

It is worth noting that despite the overall stability observed at the national level, the population of several shorebird species appears to be declining rapidly over the past decade.

### 3.4. Threats

**Figure 7:**
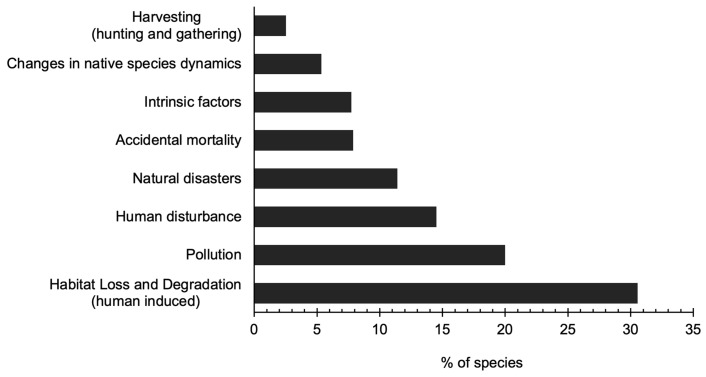
Threat analysis based on general categories [24] and BirdLife International [23,30].

We conducted a comparison of threats and potential threats to all species of shorebirds using the IUCN Red List threat categories and the database format of dominant threats. Our analysis showed that 68% of 62 shorebird species are significantly threatened by human-induced factors, including habitat loss and degradation, pollution, disturbance, and harvesting (t = 3.8, *p* = 0.003). Additionally, 11% of species face threats from natural disasters, while 8% are impacted by accidental mortality and intrinsic factors within each category. Furthermore, 5% of species are affected by changes in native species (Figure 7, Appendix B).

**Figure 8:**
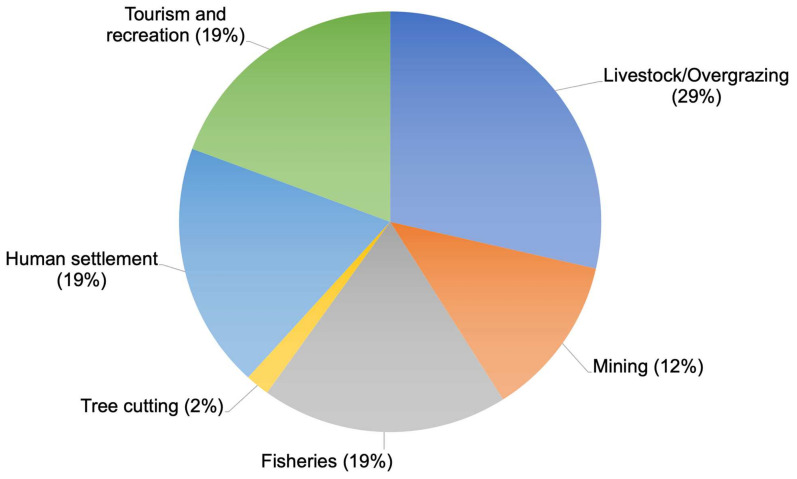
Analysis of threats to shorebirds in Mongolia.

The analysis revealed no statistical difference between threats categorized under habitat loss and degradation (D = 0.274, *p* > 0.5). However, livestock overgrazing, resulting in habitat degradation and loss, emerged as the predominant threat, accounting for 29% of the total threats to shorebirds. This was closely followed by fisheries, including entanglement with fishing line (19%), human settlement (19%), tourism and recreation (19%), mining (12%), and tree cutting (2%), which were identified as significant risks to numerous shorebird species in Mongolia (Figure 8).

**Figure 9:**
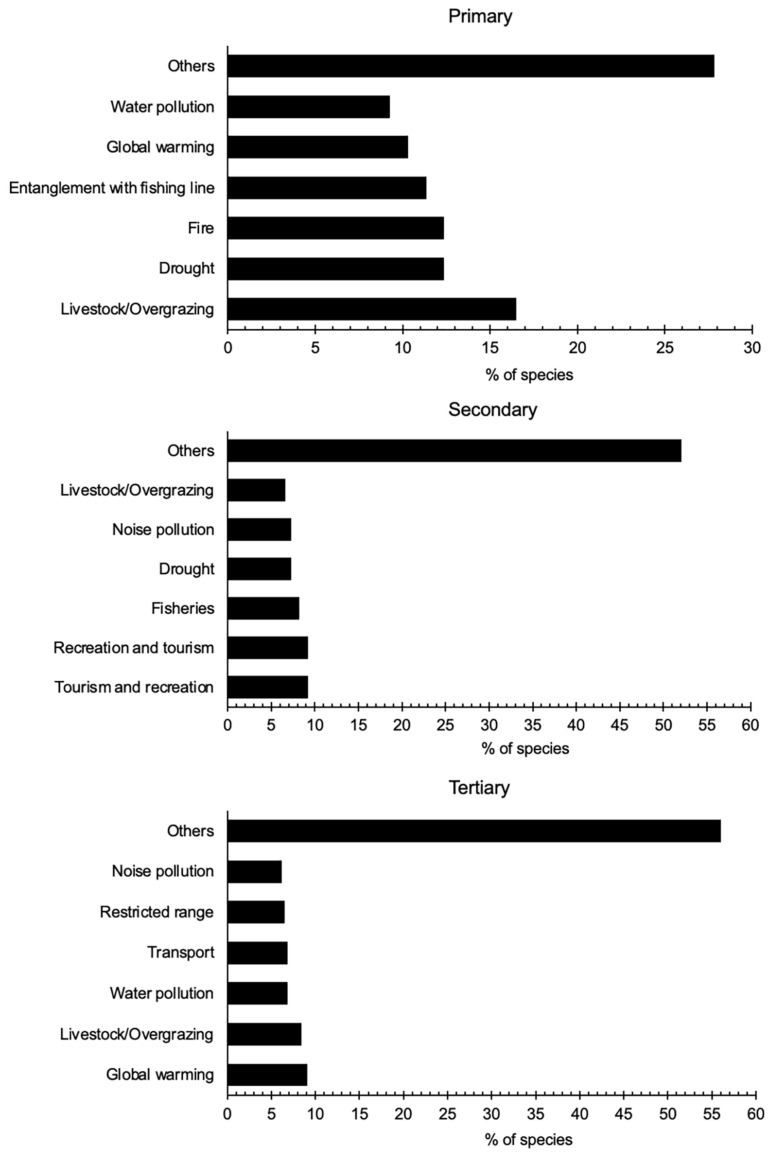
Primary, secondary, and tertiary threats to shorebirds in Mongolia.

In our analysis, we categorized threats into primary, secondary, and tertiary levels for each species, considering their relative importance and severity (Figure 9). Interestingly, we found no statistical difference (ANOVA: F_(2)_ = 13.6, *p* = 1.8) in the number of species in each of the threat ranks (primary, secondary, and tertiary), indicating that shorebird species are equally susceptible to threats across different levels. Among the identified threats, tertiary threats (n = 308) affected the highest number of species, whereas the lowest number of species were impacted by primary threats (n = 97).

When examining the number of species under each threat category, we observed significant differences (ANOVA: F_(25)_ = 2.2, *p* = 0.001). Livestock overgrazing emerged as the most pervasive threat, affecting the highest number of shorebird species (62 species). Conversely, the pollution of the environment by medicinal runoffs was found to have the lowest impact, affecting only three species.

Within the primary threat category, livestock overgrazing was identified as the most significant threatening factor, affecting 16% of shorebird species, followed closely by drought, fire, entanglement with fishing lines, global warming, and water pollution. These findings underscore the urgent need for targeted conservation efforts to mitigate the detrimental effects of these primary threats on shorebird populations.

Furthermore, the distribution of threat impacts varied across threat ranks for all shorebird species. For instance, while livestock overgrazing affected 16% of species within the primary threat category, its impact decreased to 7% and 8% within the secondary and tertiary threat categories, respectively. Similarly, drought and global warming exhibited varying degrees of impact across threat ranks, highlighting the nuanced nature of threat interactions and their differential effects on shorebird populations.

These insights into the distribution and severity of threats provide valuable guidance for prioritizing conservation strategies and interventions aimed at safeguarding shorebird populations in Mongolia and beyond.

### 3.5. Background of Shorebird Conservation

Despite the limited initiation and implementation of conservation actions for shorebirds by ministries and nature conservation institutions in Mongolia, there has been a concerted effort to protect these species within the framework of existing legislation, international conventions, and the establishment of protected areas. While specific legislative actions and legal frameworks dedicated to shorebird conservation are yet to be developed in Mongolia, various initiatives have been undertaken to safeguard these avian populations. Key actions and initiatives are presented in subsequent sections.

#### 3.5.1. Tradition and Culture of Nomadic Life

The tradition and culture of nomadic life in Mongolia are deeply intertwined with the belief systems and practices of its Buddhist population. As of 2020, approximately 87.1% of Mongolia’s total population adheres to Buddhism [34]. Within this cultural context, migratory birds, including shorebirds, hold a revered status as beings of the heavens and skies.

For Mongolian nomads, who make up a significant portion of the population, migratory birds are not merely animals but respected companions of their nomadic lifestyle. With a total of 247,870 nomadic families as of 2024 [35], these families coexist harmoniously with migratory shorebirds, welcoming their arrival in spring and bidding them farewell as they depart in autumn.

Central to this coexistence is a profound reverence for migratory creatures, stemming from deeply ingrained cultural beliefs. Nomads refrain from hunting migratory birds, viewing them as sacred beings deserving of protection. It is customary for individuals to avoid disturbing bird nests, refraining from touching eggs or chicks out of a belief that such actions may bring misfortune upon themselves and their descendants.

This longstanding tradition of respect for migratory birds serves as a potent conservation measure, even in the absence of formal legislation. The collective ethos of reverence and restraint exhibited by Mongolian nomads underscores the significance of cultural practices in safeguarding the natural world.

#### 3.5.2. Mongolian Laws and Resolutions

In Mongolia, the protection and sustainable utilization of biodiversity, including migratory birds such as shorebirds, are governed by a series of nature and wildlife-related laws and resolutions. These legislative instruments play a crucial role in safeguarding the country’s natural heritage. Some of the major laws and resolutions directly relevant to avian conservation in Mongolia include the following:

Mongolian Law on Protection of Environment (1995): This law, adopted by the Mongolian Parliament in 1995, encompasses provisions for environmental protection across various sectors. Chapters 4–7 of the law specifically address the protection and creation of databases for fauna, including birds, within the country.

Mongolian Law on Fauna (2012): Adopted on 15 May 2012, this law consists of 39 articles and four chapters. Articles 6–10 of this legislation cover important bird conservation measures, principles of protection, protection actions, and sustainable use. It has played a vital role in shorebird protection over the past decade

Mongolian Law on Regulation of Foreign Trade in Endangered Animals, Plants and Derivatives Thereof (2002): Enacted on 7 November 2002, this law regulates the trade, export, import, and re-export of scientific samples, raw materials, and live animals, including shorebirds, within Mongolia and between countries.

Mongolian Law on Specially Protected Areas (1994): Adopted on 15 November 1994, this law comprises 44 articles and eight chapters. Articles 33 and 41 specifically regulate research and conservation activities of fauna, including birds, within protected areas.

Additionally, the Mongolian Government has issued resolutions aimed at protecting rare and very rare animals, including birds. For example, Governmental Resolution 264, adopted on 5 December 2001, listed species such as the Asian Dowitcher and Black-winged Stilt under the category of rare animals. However, this resolution was invalidated in 2017, and efforts are underway to draft a new resolution.

Protected areas in Mongolia, totaling 27.2 million hectares or 17.4% of the country’s surface area, play a significant role in bird conservation. These areas include strictly protected areas, national parks, nature reserves, and natural and historical monuments. Many shorebirds breed, forage, roost, rest, and refuel within these protected areas, with some serving as important stopover sites during migration. Distribution maps created in 2011 indicate that every species of shorebird, including Breeding Visitors, Passage Migrants, and Partial Migrants, migrates through these protected areas. On average, 7.8% of the species’ range of shorebirds occurs within Mongolia’s protected areas, highlighting their importance for shorebird conservation efforts. Notable protected areas such as the Mongol Daguur Strictly Protected Area and Khalkh Numrug Protected Area play a significant role in protecting shorebirds in Mongolia.

#### 3.5.3. Conventions

Mongolia has actively engaged in international conservation efforts through various conventions and agreements, as well as through the development and implementation of national action plans and initiatives. Some of the key actions and initiatives related to shorebird conservation in the country include the following:

Convention on Biological Diversity (CBD): Mongolia became a member country of the CBD in 1993. Since then, the country has launched multiple national biodiversity action plans (NBAPs) aimed at conserving biodiversity, including migratory birds such as shorebirds.

Ramsar Convention: Mongolia signed the Ramsar Convention in 1998 and designated 11 sites as internationally important wetlands supporting migratory shorebirds. These sites serve as critical breeding, foraging, roosting, and stopover habitats for shorebirds along various flyways.

Convention on Migratory Species (CMS): Mongolia became a contracting party to the CMS in 1999. The country actively participates in CMS resolutions and declarations, particularly related to the protection of raptors and Siberian Crane. Shorebirds are listed in the CMS appendices, highlighting the international collaboration needed for their conservation.

Red Listing of Vertebrates: Mongolia initiated red listing efforts for vertebrates in 2006, leading to the publication of the first *Regional Red List for Mongolian Birds* in 2011. This publication provided crucial data and information for avian protection laws and regulations, including those pertaining to shorebirds.

Mongolian Red Book: Published in 2013 and 2016, the *Mongolian Red Book* includes assessments of the status, distribution, threats, and conservation measures for various species, including 18 wetland species of birds. While not legislatively binding, the *Mongolian Red Book* informs actions for the protection of rare and endangered species, such as the Asian Dowitcher.

Important Bird Areas (IBAs): Identified in 2009, Mongolia has 70 designated IBAs, which serve as crucial sites for bird conservation, including shorebirds. While not legally binding, IBAs provide important data and information for policymaking and legislation related to shorebird conservation.

Through these international conventions, national action plans, and initiatives, Mongolia demonstrates its commitment to the conservation of migratory birds, including shorebirds, and the protection of their habitats within the country.

## 4. Discussion

### 4.1. Species Composition, Ecology, and Conservation Status

The comprehensive analysis of shorebird species in Mongolia reveals significant advancements in our understanding of avian diversity and migration patterns over the past century. With increased field surveys and systematic data collection efforts, the number of identified shorebird species has grown substantially [5,6,7,8,9,10,11,12,31,32,33]. Today, Mongolia hosts a diverse array of shorebirds, with various migratory patterns and ecological roles within the country’s ecosystems.

The migration routes of shorebirds are intricate and span vast distances, connecting breeding grounds in northern regions with wintering grounds in southern latitudes. Mongolia serves as a crucial stopover site for many species undertaking this remarkable journey. While the East Asia–Australasia Flyway [36] is a predominant route for shorebirds breeding in Mongolia, additional flyways, including the West Asia–Africa Flyway and the Central Asia–Indian Sub-Continent Flyway, also contribute to the migration patterns observed in the country. Mongolia stands out as one of the countries boasting a high diversity of shorebird species within the East Asia–Australasian Flyway. Meanwhile, Australia hosts 37 migratory species that visit regularly [37], and 83 species utilize coastal artificial habitats along the EAAF [38].

Despite the progress made in understanding shorebird migration [39,40], there are still gaps in knowledge, particularly regarding major stopover sites and the impact of environmental changes on migratory behavior. Addressing these gaps through wide-scale migration studies will be essential for facilitating international cooperation and conserving shorebird populations across flyways.

In Mongolia, several shorebird species face significant threats, with six species classified as globally threatened by Birdlife International. These threats primarily stem from human-induced activities such as habitat loss, degradation, and disturbance. While some species are classified as Least Concern, others, like the Asian Dowitcher, are regionally or nationally threatened [10,24], underscoring the importance of conservation efforts within the country.

National and global assessments conducted by the IUCN Red List unmistakably underscore Mongolia’s significance as a crucial breeding and passage migrating site for globally threatened and Near-Threatened shorebirds traversing along the East Asian–Australasian, Central Asian, and West Asian Flyways.

### 4.2. Distribution and Species Richness

The spatial distribution of shorebird species richness throughout Mongolia mirrors broader trends in avian biodiversity, exhibiting elevated richness in the northern and eastern expanses contrasted with lower levels in the southern and western territories [10]. This distributional pattern is intricately tied to the ecological intricacies prevailing in the northern reaches, where a mosaic of diverse aquatic ecosystems thrives within the basins delineated by Namsrai et al. [19] as the Central Asian Internal Drainage Basin, Arctic Ocean, and Pacific Ocean.

The disparate availability of suitable habitats, pivotal for facilitating breeding, foraging, and roosting behaviors among shorebirds, emerges as a principal determinant shaping species richness. Within the northern territories, the coalescence of varied habitat typologies, ranging from wetlands and lakes to rivers and marshlands [18,19,41], furnishes an expansive array of resources and nesting grounds conducive to sustaining a multifarious spectrum of shorebird species.

Conversely, the southern domains, characterized by desert steppe and the formidable Gobi Desert [16,17,18], exhibit diminished species richness attributable to the paucity of water reservoirs [19,41] and the concomitant dearth of suitable shorebird habitats. The aridity prevailing in these regions, compounded by the absence of robust aquatic ecosystems, imposes constraints on both the diversity and abundance of shorebird populations. Regarding species richness (Figure 5), several wetlands within the geobotanical divisions, including the Mongol Daguur Steppe, Eastern Mongolian Plain (around Buir Lake and the northeastern part), the northern limit of Middle Khalkh Steppe, Great Lakes Depression, certain areas of the Ikh Khyangan Mountain Range, the eastern Khangai Mountain Range, and the Khuvsgul Mountain Range (specifically Darkhad Depression), emerge as key hotspots designated for the conservation of migratory and breeding shorebirds in Mongolia.

The congruence observed between the spatial distributional trends of shorebird species richness [10] and those documented for the broader avian populace in Mongolia accentuates the pivotal role played by habitat availability and complexity [16,17,18,19] in sculpting avian biodiversity. ERGO’s concerted conservation endeavors aimed at safeguarding and rehabilitating critical habitats, particularly within the northern reaches, emerge as imperative imperatives for fostering the sustenance of robust shorebird populations and preserving Mongolia’s rich avian tapestry.

### 4.3. Population Trends

The global trajectory of shorebird populations within Mongolia highlights significant concerns regarding declines observed across diverse regions worldwide. Studies conducted in locations such as Bahrain [42], Australia [43], and India [44], in conjunction with global-scale analyses [45,46], consistently indicate notable reductions in shorebird populations, largely attributed to factors such as habitat loss, degradation, climate variability, and human-induced activities.

Within Mongolia, while the overarching analysis of national population trends indicates a semblance of stability in the majority of shorebird populations (61%), a notable fraction remains enshrouded in uncertainty (34%) or, concerning, shows signs of decline (5%). However, apprehensions escalate as rapid declines manifest in select species, exemplified by the diminishing presence of breeding pairs such as the Asian Dowitcher, Black-Tailed Godwit, Marsh Sandpiper (*Tringa stagnatilis*), and Ruff over the past two decades. The imperative for systematic population surveillance and robust data acquisition protocols across pivotal wetland habitats in Mongolia becomes starkly apparent.

These findings resonate with global scholarship, underlining the pivotal role of migration-associated stressors, including habitat degradation and deterioration at stopovers or wintering grounds [47,48], alongside the amplifying impact of climate perturbations [47], in precipitating worldwide shorebird declines.

To effectively redress these challenges and curtail population downturns, a concerted, multi-pronged approach is imperative. This entails the implementation of comprehensive conservation strategies, encompassing habitat rehabilitation initiatives and the meticulous monitoring of shorebird populations. Moreover, fostering international synergies and forging collaborative research alliances assumes paramount importance, facilitating a nuanced comprehension of the multifaceted drivers dictating shorebird population dynamics and, correspondingly, the formulation of targeted conservation interventions.

### 4.4. Threats

The comprehensive threat assessment conducted for shorebirds in Mongolia illuminates the pervasive influence of human-induced perils on these avian species, with a staggering 68% of species flagged as imperiled by factors encompassing habitat deterioration, pollution, disturbance, and exploitation. Additionally, threats stemming from natural calamities, inadvertent mortality, intrinsic ecological shifts, and alterations in indigenous species dynamics compound the challenges confronting shorebird conservation. Notably, recreational pursuits, tourism influxes, and entanglement with fishing paraphernalia emerge as prominent hazards to shorebird populations in Mongolia, as depicted in Figure 4 (refer to Appendix B).

These findings resonate with threat analyses conducted in analogous contexts, such as North America [48], and on a global scale [47], which similarly pinpoint anthropogenic activities as pivotal drivers of population downturns. In coastal regions across Asia, supplementary perils like mega-tsunamis, volcanic eruptions, and the conversion of coastal habitats exacerbate the challenges confronting shorebird conservation efforts.

The compounded repercussions of concurrent threats, exemplified by phenomena such as overgrazing, drought, wildfires, alterations in indigenous species dynamics, and human encroachment, exact a particularly heavy toll on shorebird populations, accentuating the exigency of simultaneously addressing multifaceted challenges. The lamentable disappearance of breeding pairs, as observed in species like the Asian Dowitcher [49], attributed to habitat degradation and overgrazing, underscores the pressing imperative for concerted conservation initiatives across Mongolia’s wetland landscapes.

Moreover, the deleterious impact of human-induced fires in steppe and forest-steppe ecosystems during the spring and early summer months, affecting regions in eastern and northeastern Mongolia, as well as southeastern Russia [50], underscores the precarious vulnerability of shorebird nesting habitats. Incidents of such fires have been observed to obliterate nests containing eggs of species such as the Marsh Sandpiper, Black-Tailed Godwit, Asian Dowitcher, Pied Avocet, (*Recurvirostra avosetta*), Common Snipe, (*Gallinago gallinago*), and Oriental Plover, particularly in open marshy areas characterized by dense vegetation and reeds.

Mitigating these myriad threats necessitates prioritizing strategies aimed at ameliorating habitat degradation and loss, mitigating pollution and disturbance, and fostering sustainable land management practices. Furthermore, proactive measures to alleviate the impact of natural disasters, including storms and flooding, assume critical importance in fortifying the resilience of shorebird populations, particularly those already imperiled.

In summary, the formulation and execution of comprehensive conservation frameworks, underpinned by collaborative endeavors involving governmental entities, conservation agencies, and local stakeholders, stand as imperative imperatives to safeguard shorebird populations and their habitats, both within Mongolia and on a global scale. By addressing the underlying drivers of threats and instituting efficacious conservation interventions, the enduring viability of these emblematic avian species can be assured for posterity.

## 5. Strategies for Enhancing Shorebird Conservation in Mongolia: Addressing Weaknesses and Building Capacity

Efforts to protect shorebirds in Mongolia benefit from a robust framework of legislative measures, international conventions, and national initiatives ([19,33], https://legalinfo.mn, accessed on 20 April 2024). These encompass laws governing environmental protection, regulations pertaining to the foreign trade of endangered species, and the establishment of protected areas. Furthermore, Mongolia’s active participation in conventions such as the CBD [51,52], Ramsar [33], and CMS [33] underscores its dedication to global biodiversity conservation endeavors.

Looking ahead, sustained collaboration among governmental bodies, conservation organizations, and researchers will be indispensable for safeguarding shorebird populations and their habitats in Mongolia and beyond. By addressing knowledge gaps, implementing targeted conservation measures, and advocating for sustainable practices, we can ensure the enduring viability of these emblematic migratory species.

Addressing the existing shortcomings in conservation legislation and initiatives pertaining to shorebirds in Mongolia is paramount to ensuring the enduring protection of these invaluable species. Several potential strategies aimed at remedying these weaknesses are outlined as follows:

1. Economic Incentives: Introducing economic incentives could prove instrumental in galvanizing conservation efforts. Mechanisms such as funding schemes or tax incentives for landowners who undertake habitat restoration or conservation practices conducive to shorebirds could be explored. Moreover, fostering public–private partnerships could unlock additional funding streams, leveraging the interests of businesses with a stake in conservation.
2. Enhanced Collaboration: Strengthening collaboration and communication across various stakeholders is pivotal. Facilitating robust engagement between researchers, policymakers, and conservation practitioners ensures that policy decisions and conservation actions are informed by sound scientific evidence. Establishing interdisciplinary working groups or advisory committees specifically dedicated to shorebird conservation could serve as conduits for effective collaboration.
3. Data Collection and Monitoring: Investing in comprehensive monitoring programs is essential for gathering accurate data on shorebird populations, distribution, and habitat utilization. This could entail capacity-building initiatives to train local scientists and volunteers in standardized monitoring protocols. Leveraging technological advancements such as satellite tracking and remote sensing can enhance data collection over vast geographic expanses.
4. Capacity Building: Building capacity among local stakeholders is crucial for sustained conservation efforts. Providing training and support to local scientists, conservation organizations, and community stakeholders through workshops, internships, and exchange programs fosters expertise in shorebird conservation best practices.
5. Integration with Legislation: Integrating shorebird conservation assessments into existing legislation and policies is imperative. Updating legal frameworks, such as the Mongolian Laws on Protection of Environment and Fauna, to incorporate specific protections for shorebirds and their habitats based on scientific assessments and international agreements is essential.
6. Red List Updates: Conducting regular updates of the *Regional Red List for Mongolian Birds* ensures that conservation priorities remain aligned with the most current data. Periodic assessments of shorebird populations utilizing standardized criteria and methodologies, with input from national and international experts, are essential for informed decision-making.
7. Institutional Strengthening: Strengthening institutional capacity within government agencies responsible for environmental management and conservation is vital. This could involve allocating additional resources, enhancing coordination between agencies, and delineating clear mandates for shorebird conservation within governmental structures.

By addressing these identified weaknesses and implementing targeted strategies for shorebird conservation, Mongolia can make significant strides toward safeguarding these emblematic species and their habitats for the benefit of future generations.

## 6. Conclusions

In summary, the review on the conservation status of shorebirds in Mongolia provides a detailed examination of the ecological significance of the country as a crucial breeding and migration site for 62 shorebird species, including several globally threatened ones. By analyzing population trends, this document reveals that while most shorebird species in Mongolia exhibit stable trends at the national level, a significant proportion globally are experiencing declines. This review identifies human-induced factors such as habitat loss, pollution, disturbance, and harvesting as major threats to 69% of shorebird species, underscoring the urgent need for targeted conservation actions.

Despite the absence of specific conservation plans dedicated to shorebirds in Mongolia, this review highlights the existence of general conservation measures, including environmental and fauna protection laws, regulations on endangered species trade, and the establishment of protected areas under governmental resolutions. Mongolia’s participation in international conventions such as the Convention on Biological Diversity, Ramsar, and Migratory Species further demonstrates its commitment to biodiversity conservation.

This review emphasizes the importance of addressing key threats such as habitat loss and degradation to safeguard shorebird populations in Mongolia. By compiling the most recent species list and assessing the current status of shorebirds’ ecology and conservation, this document aims to facilitate the prioritization of conservation actions for the future. By enhancing global awareness and understanding of the conservation challenges faced by shorebirds in Mongolia, our review advocates for collaborative efforts to ensure the long-term survival of these vital avian species.

## Data Availability

Not applicable.

## Appendix A

**Table A1:** Species list, ecology, and conservation status of shorebirds of Mongolia.

No	Family Name	No	Common Name	Scientific Name	Regional Status	Mongolia IUCN Status	Global IUCN Status	Global Population Trend	Mongolia Population Trend	CMS
1	Haematopodidae	1	Eurasian Oystercatcher	*Haematopus ostralegus*	VA1	NA	NT	Decreasing	Unknown	II
2	Burhinidae	2	Eurasian Stone-curlew	*Burhinus oedicnemus*	VA2	NA	LC	Decreasing	Unknown	II
3	Recurvirostridae	3	Black-winged Stilt	*Himantopus himantopus*	BV1/2; PsM1/2	LC	LC	Increasing	Stable	II
		4	Pied Avocet	*Recurvirostra avosetta*	BV1/2; PsM1/2	LC	LC	Unknown	Stable	II
4	Charadriidae	5	Northern Lapwing	*Vanellus vanellus*	BV3; PsM3/4	LC	NT	Decreasing	Stable	II
		6	Gray-headed Lapwing	*Vanellus cinereus*	BV1; PsM1/2	NA	LC	Decreasing	Unknown	II
		7	Sociable Lapwing	*Vanellus gregarius*	VA5	NA	CR	Decreasing	Unknown	I
		8	Pacific Golden Plover	*Pluvialis fulva*	PsM3/4	LC	LC	Decreasing	Stable	II
		9	Gray Plover	*Pluvialis squatarola*	PsM2	LC	LC	Decreasing	Stable	II
		10	Common Ringed Plover	*Charadrius hiaticula*	PsM1	DD	LC	Decreasing	Unknown	II
		11	Long-billed Plover	*Charadrius placidus*	VA1	NA	LC	Decreasing	Unknown	II
		12	Little Ringed Plover	*Charadrius dubius*	BV2/3; PsM3	LC	LC	Stable	Stable	II
		13	Kentish Plover	*Charadrius alexandrinus*	BV3/4; PsM4	LC	LC	Decreasing	Stable	II
		14	Mongolian/Siberian Sandplover	*Charadrius mongolus*	PsM2	DD	EN	Decreasing	Unknown	II
		15	Greater Sandplover	*Charadrius leschenaultii*	BV2; PsM3	LC	LC	Decreasing	Stable	II
		16	Oriental Plover	*Charadrius veredus*	BV2/3; PsM2/3	LC	LC	Unknown	Unknown	II
		17	Eurasian Dotterel	*Eudromias morinellus*	BV1; PsM1	LC	LC	Decreasing	Unknown	II
5	Rostratulidae	18	Greater Painted-snipe	*Rostratula benghalensis*	VA1	NA	LC	Decreasing	Unknown	
6	Scolopacidae	19	Whimbrel	*Numenius phaeopus*	PsM1/2	LC	LC	Decreasing	Stable	II
		20	Little Curlew	*Numenius minutus*	PsM2/3	LC	LC	Stable	Stable	II
		21	Far Eastern Curlew	*Numenius madagascariensis*	PsM1	LC	EN	Decreasing	Decreasing	I
		22	Eurasian Curlew	*Numenius arquata*	BV1; PsM2/3	LC	NT	Decreasing	Stable	II
		23	Bar-tailed Godwit	*Limosa lapponica*	PsM1	LC	NT	Decreasing	Decreasing	II
		24	Black-tailed Godwit	*Limosa limosa*	BV1; PsM3	LC	NT	Decreasing	Stable	II
		25	Ruddy Turnstone	*Arenaria interpres*	PsM2/3	LC	LC	Decreasing	Stable	II
		26	Great Knot	*Calidris tenuirostris*	VA1	NA	EN	Decreasing	Stable	I
		27	Red Knot	*Calidris canutus*	PsM1	LC	NT	Decreasing	Unknown	II
		28	Ruff	*Calidris pugnax*	BV1; PsM2/3	LC	LC	Decreasing	Stable	II
		29	Broad-billed Sandpiper	*Calidris falcinellus*	PsM2/3	LC	LC	Decreasing	Stable	II
		30	Sharp-tailed Sandpiper	*Calidris acuminata*	PsM2/3	LC	VU	Decreasing	Stable	II
		31	Curlew Sandpiper	*Calidris ferruginea*	PsM3/4	LC	NT	Decreasing	Stable	II
		32	Temminck’s Stint	*Calidris temminckii*	PsM3/4	LC	LC	Unknown	Stable	II
		33	Long-toed Stint	*Calidris subminuta*	PsM2/3	LC	LC	Unknown	Stable	II
		34	Red-necked Stint	*Calidris ruficollis*	PsM3/4	LC	NT	Decreasing	Stable	II
		35	Sanderling	*Calidris alba*	PsM1/2	LC	LC	Unknown	Stable	II
		36	Dunlin	*Calidris alpina*	PsM1/2	LC	LC	Decreasing	Stable	II
		37	Baird’s Sandpiper	*Calidris bairdii*	VA1	NA	LC	Stable	Unknown	II
		38	Little Stint	*Calidris minuta*	PsM2	LC	LC	Increasing	Stable	II
		39	Buff-breasted Sandpiper	*Calidris subruficollis*	VA1	NA	NT	Decreasing	Unknown	I
		40	Pectoral Sandpiper	*Calidris melanotos*	VA2	NA	LC	Stable	Unknown	II
		41	Asian Dowitcher	*Limnodromus semipalmatus*	BV1/2; PsM2	VU	NT	Decreasing	Decreasing	II
		42	Long-billed Dowitcher	*Limnodromus scolopaceus*	VA1	NA	LC	Unknown	Unknown	II
		43	Eurasian Woodcock	*Scolopax rusticola*	BV2; PsM2/3	LC	LC	Stable	Stable	II
		44	Jack Snipe	*Lymnocryptes minimus*	PsM1	DD	LC	Stable	Unknown	II
		45	Solitary Snipe	*Gallinago solitaria*	BV1; PrM1	LC	LC	Stable	Stable	II
		46	Pintailed Snipe	*Gallinago stenura*	BV1; PsM2	LC	LC	Unknown	Stable	II
		47	Swinhoe’s Snipe	*Gallinago megala*	BV1; PsM1/2	LC	LC	Unknown	Stable	II
		48	Common Snipe	*Gallinago gallinago*	BV2/3; PsM3/4	LC	LC	Decreasing	Stable	II
		49	Terek Sandpiper	*Xenus cinereus*	PsM1/2	LC	LC	Decreasing	Stable	II
		50	Red-necked Phalarope	*Phalaropus lobatus*	PsM2/3	LC	LC	Decreasing	Unknown	II
		51	Red Phalarope	*Phalaropus fulicarius*	VA3	NA	LC	Unknown	Stable	II
		52	Common Sandpiper	*Actitis hypoleucos*	BV2; PsM3	LC	LC	Decreasing	Stable	II
		53	Green Sandpiper	*Tringa ochropus*	BV1; PsM2/3	LC	LC	Increasing	Stable	II
		54	Wandering Tattler	*Tringa incana*	VA2	NA	LC	Stable	Unknown	II
		55	Gray-tailed Tattler	*Tringa brevipes*	PsM1/2	NA	NT	Decreasing	Unknown	II
		56	Common Redshank	*Tringa totanus*	BV3; PsM3	LC	LC	Unknown	Stable	II
		57	Marsh Sandpiper	*Tringa stagnatilis*	BV1/2; PsM3	LC	LC	Decreasing	Stable	II
		58	Wood Sandpiper	*Tringa glareola*	BV1; PsM2/3	LC	LC	Stable	Stable	II
		59	Spotted Redshank	*Tringa erythropus*	PsM2/3	LC	LC	Stable	Stable	II
		60	Common Greenshank	*Tringa nebularia*	PsM2/3	LC	LC	Stable	Stable	II
7	Glareolidae	61	Collared Pratincole	*Glareola pratincola*	VA1	NA	LC	Decreasing	Unknown	II
		62	Oriental Pratincole	*Glareola maldivarum*	BV1?; PsM1	DD	LC	Decreasing	Unknown	II

## Appendix B

**Table A2:** Species list of shorebirds and threats by IUCN Red List. Dark red represents the primary threats, pink shows the secondary threats, and light gray represents the tertiary threats.

No	Common Name	1. Habitat Loss and Degradation (Human Induced)						3. Harvesting (Hunting and Gathering)		4. Accidental Mortality		6. Pollution				7. Natural Disasters			8. Changes in Native Species Dynamics		9. Intrinsic Factors				10. Human Disturbance		
		1.1.4. Livestock/Overgrazing	1.3.1. Mining	1.3.2. Fisheries	1.3.3. Tree Cutting	1.4.2. Human Settlement	1.4.3. Tourism and Recreation	3.2. Medicine	3.5. Cultural, Scientific, and Leisure Activities	4.1.1.3. Entanglement with Fishing Line	4.2. Collision	6.1.1. Global Warming	6.2. Land Pollution	6.3. Water Pollution	6.3.10. Noise Pollution	7.1. Drought	7.2. Storms and Flooding	7.3. Temperature Extremes	8.1. Competitors	8.3. Prey and Food Base	9.2. Poor Recruitment and Reproduction	9.3. High Juvenile Mortality	9.5. Low Densities	9.9. Restricted Range	10.1. Recreation and Tourism	10.4. Transport	10.5. Fire
1	Sociable Lapwing																										
2	Mongolian/Siberian Sandplover																										
3	Far Eastern Curlew																										
4	Great Knot																										
5	Sharp-tailed Sandpiper																										
6	Eurasian Oystercatcher																										
7	Northern Lapwing																										
8	Eurasian Curlew																										
9	Bar-tailed Godwit																										
10	Black-tailed Godwit																										
11	Red Knot																										
12	Curlew Sandpiper																										
13	Red-necked Stint																										
14	Buff-breasted Sandpiper																										
15	Asian Dowitcher																										
16	Gray-tailed Tattler																										
17	Eurasian Stone-curlew																										
18	Black-winged Stilt																										
19	Pied Avocet																										
20	Gray-headed Lapwing																										
21	Pacific Golden Plover																										
22	Gray Plover																										
23	Common Ringed Plover																										
24	Long-billed Plover																										
25	Little Ringed Plover																										
26	Kentish Plover																										
27	Greater Sandplover																										
28	Oriental Plover																										
29	Eurasian Dotterel																										
30	Greater Painted-snipe																										
31	Whimbrel																										
32	Little Curlew																										
33	Ruddy Turnstone																										
34	Ruff																										
35	Broad-billed Sandpiper																										
36	Temminck’s Stint																										
37	Long-toed Stint																										
38	Sanderling																										
39	Dunlin																										
40	Baird’s Sandpiper																										
41	Little Stint																										
42	Pectoral Sandpiper																										
43	Long-billed Dowitcher																										
44	Eurasian Woodcock																										
45	Jack Snipe																										
46	Solitary Snipe																										
47	Pin-tailed Snipe																										
48	Swinhoe’s Snipe																										
49	Common Snipe																										
50	Terek Sandpiper																										
51	Red-necked Phalarope																										
52	Red Phalarope																										
53	Common Sandpiper																										
54	Green Sandpiper																										
55	Wandering Tattler																										
56	Common Redshank																										
57	Marsh Sandpiper																										
58	Wood Sandpiper																										
59	Spotted Redshank																										
60	Common Greenshank																										
61	Collared Pratincole																										
62	Oriental Pratincole																										
